# Primary Effusion Lymphoma Presenting As Parapneumonic Pleural Effusion

**DOI:** 10.7759/cureus.26794

**Published:** 2022-07-12

**Authors:** Rahul Gujarathi, Narsimha Candula, Venu Chippa, Meet Kadakia, Ahmad Alkhasawneh

**Affiliations:** 1 Hospital Medicine, University of Florida Health, Jacksonville, USA; 2 internal Medicine, St Vincent Medical Center, Evansville, USA; 3 Hematology/Oncology, University of Florida College of Medicine – Jacksonville, Jacksonville, USA; 4 Pathology, University of Florida College of Medicine – Jacksonville, Jacksonville, USA

**Keywords:** hhv-8, hiv aids antiretrovial therapy, rare cause of pleural effusion, immunocompromised patient, non hodgkin's lymphoma

## Abstract

Primary effusion lymphoma (PEL) is a rare form of high-grade non-Hodgkin's lymphoma that usually occurs in patients with compromised immunity or human immunodeficiency virus (HIV) infection. PEL is a B-cell lymphoma that principally presents as effusions without a tumor mass. We present a case of a 56-year-old African-American male with a medical history of HIV admitted to the hospital with right lung lower lobe pneumonia and parapneumonic effusion. Thoracentesis and pleural fluid cytology led to the diagnosis of PEL. He received treatment with chemotherapy and antiretroviral therapy (ART). The emphasis is to investigate immunocompromised patients presenting with pleural effusion for PEL, as it is a rare ailment with a high mortality rate.

## Introduction

A primary effusion lymphoma (PEL) is a rare form of high-grade non-Hodgkin's lymphoma (NHL)that usually occurs in patients with compromised immunity or HIV infection. As per the Centers for Disease Control and Prevention (CDC) in the United States of America (USA), approximately 1.2 million people have HIV. PEL is a rare HIV-associated NHL with a predilection for body cavities (pleural space, peritoneum, and pericardium). PEL is strongly causally related to Human herpes virus-8 (HHV8), also known as Kaposi’s sarcoma herpes virus (KSHV), and its presence has been incorporated as a diagnostic criterion for PEL [1]. PEL accounts for less than 4% of all HIV-related lymphoma [2-4]. The incidence of AIDS-related NHL and primary central nervous system lymphoma has significantly decreased with ART since the 1990s [3]. Though PEL is commonly seen with HIV, it is also reported in people after solid organ transplantation, receiving immunosuppression therapy [5-8]. About 2% of HIV-positive individuals develop pleural effusion; the most common causes are parapneumonic effusions, empyema, tuberculosis, Kaposi sarcoma, and rarely Pneumocystis carinii infection [9]. Pleural effusion in HIV patients should be thoroughly worked up for possible infections and malignancies like lymphoma as it has different treatment and outcomes.

## Case presentation

A 56-year-old African-American male with a medical history of HIV not on ART, an active smoker presented to the emergency department with complaints of productive cough, shortness of breath, and associated right-sided pleuritic chest pain for the last four weeks. The patient was admitted to the hospital four weeks ago for right lower lobe pneumonia and was discharged on oral Levofloxacin for five days, which he was not compliant with. The patient returned back to the hospital with no improvement in his symptoms. Upon further elaboration of history, he also reported losing approximately 25 pounds weight in the prior six months and having night sweats for the last three to four weeks.

A physical exam was pertinent for a cachectic male patient in mild respiratory distress. Has oral candidiasis on inspection and decreased breath sounds on the right side compared to the left side on auscultation. Laboratory analysis revealed leukopenia with a white blood cell count of 2.91 thousand per cubic mm, anemia with hemoglobin of 10.9 g/dL, and a platelet count of 209 thousand per cubic mm. His creatinine was 0.97 mg/dL, sodium was 131 mmol/L, and CD4 count was 128 cells/µL.

**Figure 1:**
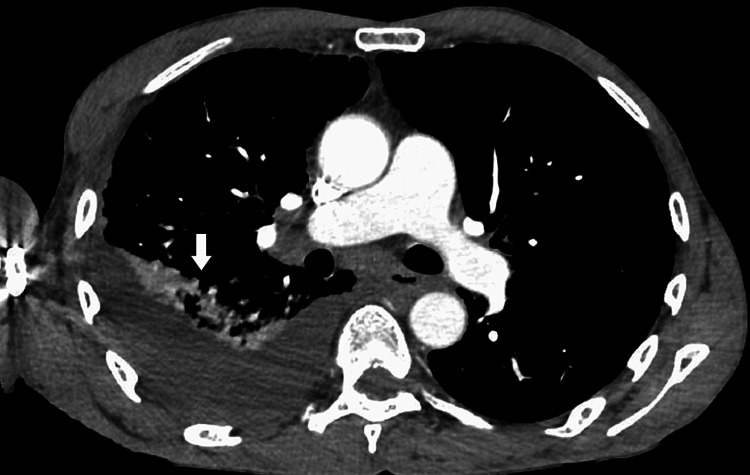
CT scan of the chest with contrast (axial view) showing right-sided pleural effusion

**Figure 2:**
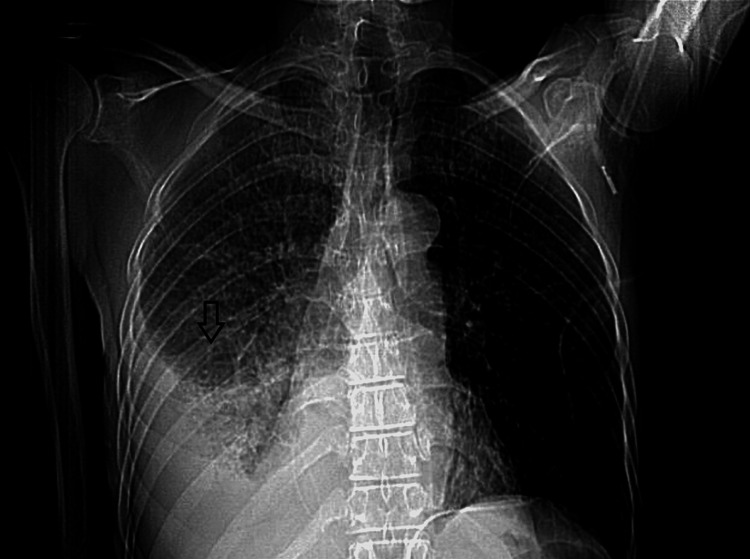
CT scan of the chest with contrast (coronal view) showing right-sided pleural effusion

Chest x-ray revealed right-sided pleural effusion. Computed tomography (CT) of the chest revealed right lung lower lobe Pneumonia as shown in the axial view (Figure 1) and large right-sided pleural effusion as shown in the coronal view (Figure 2).

 

The patient was admitted and started on antibiotics (Cefepime intravenously 2 g every eight hours and Zithromax orally 500 mg every 24 hours) for right-sided pneumonia and parapneumonic effusion. Subsequently, he underwent diagnostic and therapeutic right-sided thoracentesis. Pleural fluid analysis revealed the exudative etiology of pleural effusion. Cytology of pleural fluid was sent given his type-B symptoms, and the results were consistent with PEL. Pleural fluid flow cytometry revealed a monoclonal population compatible with lymphoma of B-cell origin and consistent with the PEL. Pleural fluid immuno-stain results were positive for markers CD30, MUM-1 (Multiple Myeloma-1), Ki-67 (proliferation marker), Kappa- ISH (in-situ Hybridization), and Epstein-Barr virus-encoded RNA (EBER). Pleural fluid immuno-stain results were negative for markers such as CD20, PAX-5, CD138, and Lambda-ISH.

**Figure 3:**
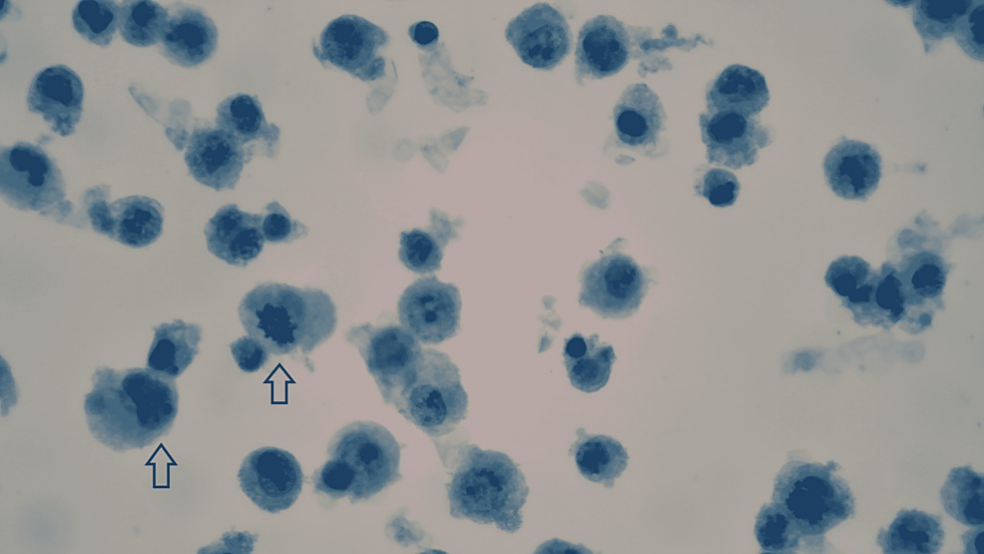
Pleural effusion shows numerous neoplastic lymphoid cells with plasmablastic morphology (Pap stain, 630x)

**Figure 4:**
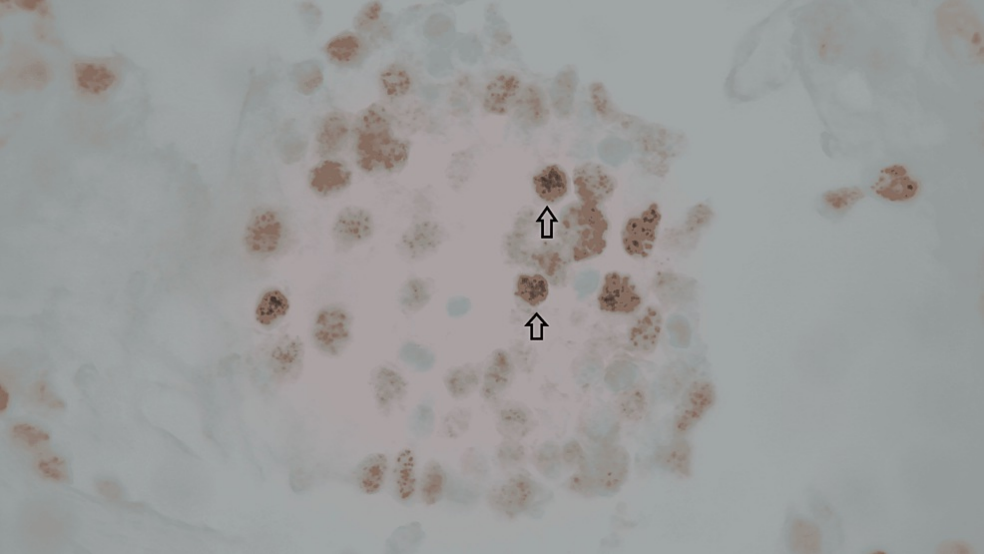
The cells are immunoreactive for HHV-8 (Immuno-histochemical stain, 630x)

Figure 3 shows the neoplastic lymphoid cells of PEL with plasmablastic morphology on the Pap stain and Figure 4 shows the PEL cells immuno-reactive for HHV-8 on the immuno-histochemical stain.

The patient was promptly started on chemotherapy with EPOCH regimen (Etoposide, Prednisolone, Oncovin (Vincristine), Cyclophosphamide, and Hydroxydaunorubicin (Adriamycin)). He was also started on ART (combination of bictegravir, emtricitabine, and tenofovir alafenamide) for his AIDS. The patient completed six cycles of chemotherapy treatment without significant side effects except for developing peripheral neuropathy during his cycle four chemotherapy, as a result of which Vincristine was discontinued. The patient was admitted again two months after completion of chemotherapy with acute hypoxic respiratory failure and multi-organ dysfunction leading to his death in the hospital.

## Discussion

PEL is a rare B-cell malignancy that most often occurs in immunocompromised patients, such as HIV-infected individuals and patients receiving organ transplantation. The main characteristic of PEL is neoplastic effusions in body cavities without detectable tumor masses [10]. This entity is classified as a mature B-cell neoplasm in the 2001 World Health Organization (WHO) classification of tumors of hematopoietic and lymphoid tissues. PEL is universally associated with HHV-8 [1]. HHV-8 seems to play a major role in the pathogenesis of PEL by promoting proliferation and impairing apoptosis [11-13].

PEL originates on the serosal surfaces, including the pleura (60% to 90%), peritoneum (30% to 60%), pericardium (up to 30%), joint spaces, and very rarely, the meninges [12,14,15]. Extra-cavitary PEL is a clinical variant of PEL that characterizes by solid tumor lesions without malignant serous fluid, and it accounts for approximately one-third of all cases of PEL [16,17].

Immunohistochemical staining for the viral gene product, latency-associated nuclear antigen (LANA-1), is the most common method for detecting HHV-8 positivity [18]. Over 90% of PEL cases demonstrate expression of CD45, and plasma cell-related markers such as epithelial membrane antigen, CD30, CD38, CD71, and CD138 are usually present [19]. B-cell antigens (CD19, CD20, CD79a) and T-cell-associated antigens are frequently negative but can be seen in a few cases [20]. The malignant cells display a range of morphologic appearances, from large plasmablastic or immunoblastic cells to those with more anaplastic features, and few cells can resemble Reed-Sternberg cells [18].

The differential diagnosis of PEL includes systemic lymphomas with secondary involvement of the body fluid (secondary effusion), lymphomas that develop due to chronic pyothorax (pyothorax-associated lymphoma), and extra-nodal variants of various subtypes of lymphoma (such as extra-nodal large cell lymphoma). PEL is distinguished from these other types of lymphoma by its HHV-8 positivity [19]. Kaposi sarcoma inflammatory cytokine syndrome (KICS) is a relatively new syndrome described in patients co-infected with HIV, and KSHV also falls under the differential diagnosis for PEL [21].

PEL can cause local destruction and consistently has a poor prognosis without treatment [14]. The median overall survival (OS) after PEL diagnosis, without treatment, is approximately two to three months [20]. Even with aggressive chemotherapy, the median OS extended on average to only six months [22,23]. Treatment approaches for PEL include ART, cytotoxic chemotherapy, radiation therapy, and their combinations. Most clinicians advocate the administration of both ART and combination chemotherapy as initial treatment. Case reports have illustrated that some patients with PEL can obtain Complete Remission (CR) with ART alone [4,12,15,19]. However, this approach is reserved for patients with poor performance status or other comorbidities that would preclude using combination chemotherapy. When choosing the ART regimen, it is essential to consider overlapping interference or toxicities of antiretroviral medications with chemotherapeutic medications that may be used in the future.

There is a lack of data to guide treating patients with PEL. Dose-adjusted EPOCH (Cyclophosphamide, Doxorubicin, Etoposide, Vincristine, Prednisone) or CHOP (Cyclophosphamide, Doxorubicin, Vincristine, Prednisone) is used in the treatment of PEL. CODOX-M (Cyclophosphamide, Vincristine, Doxorubicin, high-dose Methotrexate) with IVAC (Ifosfamide, Cytarabine, Etoposide, and intrathecal Methotrexate), also called the Magrath regimen, has been used for tumors with a very high growth fraction. This regimen has more toxicity and is reserved for patients with a few comorbidities and good functional status. Rituximab is incorporated into the regimen [12] in rare cases of PEL composed of B-lymphocyte antigen (CD20)-positive cells. The efficacy of consolidation with hematopoietic cell transplantation (HCT) for PEL is unknown. In one study, OS was 75%, and two-year progression-free survival was 50% in four patients who underwent autologous HCT after achieving a complete response with anthracycline-based combination chemotherapy [24]. Radiation therapy to the body cavity of origin may provide symptomatic relief when chemotherapy is not feasible, or the patient has failed other treatment regimens [25]. There is even less evidence to guide the treatment of patients with PEL who are negative for HIV, as this is a highly uncommon patient population. Since PEL is so rare, there are very few retrospective series and no prospective trials in this patient group.

## Conclusions

PEL is an aggressive and rare lymphoma with high mortality, which can present as pleural effusion. Assessing for “B” symptoms, including fever, night sweats, and unintentional weight loss, is vital during history taking process. While it is relevant to evaluate for and treat the infection first, be vigilant not to miss alternative inflammatory, autoimmune, or malignant etiologies. This case report encourages clinicians to consider a broad differential, especially in HIV patients diagnosed with exudative pleural effusion, to investigate further with pleural fluid cytology. Diagnostic suspicion for PEL leads to early identification of the disease and initiation of treatment promptly. Emphasis must be placed on an expedited diagnosis and early enrollment in clinical trials.
